# A two-group kinetic wealth model with wealth-gap drift and non-Maxwellian kernels

**DOI:** 10.1371/journal.pone.0336043

**Published:** 2025-11-06

**Authors:** Rongmei Sun

**Affiliations:** School of Mathematics, Southwestern University of Finance and Economics, Chengdu, China; Khalifa University of Science and Technology, UNITED ARAB EMIRATES

## Abstract

This paper applies statistical mechanics to investigate wealth distribution in binary interactions between two groups of agents. Using an exchange rule with non-zero expected random variables and non-Maxwellian collision kernels, we consider the case that wealth distribution is affected by the wealth replacement rate, trading rate, market risk and the proportion of steady-state wealth distributions of two groups of agents. The decrease of market risk and the increase of the wealth replacement rate and trading rate are conducive to the equalization of wealth distribution, and high proportion of steady-state wealth distributions of two groups of agents narrows disparities in group 1 but worsens them in group 2 under certain conditions. We verify our conclusions by numerical experiments.

## Introduction

The wealth distribution is not only a core indicator for evaluating the fairness of social wealth allocation, but also a key factor influencing economic growth and social stability. Therefore, it is crucial to investigate the evolution and determinants of wealth distributions. Combining economic theory and real data analysis, scholars conclude that taxation [1], education [2], personal savings [3], pension insurance [4] and inherited wealth [5] influence the wealth distribution. We employ the statistical mechanics approach to discuss the evolution and influencing factors of wealth distribution.

The rarefied gas kinetic theory is one of the important components of the theoretical physics. In the 19th century, using a non-negative function *f*(*r*, *v*, *t*) ($r\in R^{3}$ and $v\in R^{3}$ represent the position and velocity of the gas particles at time *t*, respectively) as the density function of rarefied gas particles, Maxwell [6] obtained the steady-state distribution of gas particles. Boltzmann [7] derives the integral-differential equation for the evolution of the distribution function of gas particles. This integral-differential equation is named the Boltzmann equation

$$\frac{\partial f(r,v,t)}{\partial t}=-v[f(r+\Delta r,v,t)-f(r,v,t)]+Q(f,f)(r,v,t),$$ (1)

where Δr stands for the change of position of the gas particle, and −v[f(r  +  Δr,v,t) − f(r,v,t)] measures the influence of the movement of gas particles on distribution. *Q*(*f*, *f*)(*r*, *v*, *t*) is a collision operator that describes the impact of energy exchange between particles on the distribution. Carleman [8] investigates the theory of spatial homogeneity and proves the existence and uniqueness of the solution to (1) under certain conditions. When the density variation of gas particles no longer depends on the position *r* of the particles in space, but rather on the collisions between particles, Eq (1) is simplified to


$$\frac{\partial f(r,v,t)}{\partial t}=-Q(f,f)(v,t).$$

Due to the complexity of the collision operators, in general, the Boltzmann equation cannot be solved directly. Two methods are commonly used: one is converting the Boltzmann equation into the Fokker-Planck equation and obtaining its steady-state solution under certain assumptions (see [9–11]), and the other is performing numerical simulations on kinetic equations. Pareschi and Toscani [12] elaborate on the applications of multi-agent kinetic equations across economics, social sciences and biology, while detailing the construction of efficient simulation algorithms via the Monte Carlo method. This method provides valuable references for the numerical simulation of multi-agent systems.

In recent decades, scholars have applied the rarefied gas kinetic theory to multiple disciplines, especially in the socioeconomic field. Pareto [13] discovers that the wealth distribution in western countries follows a power-law distribution, that is, when t→∞, the wealth distribution satisfies


$$f_{\infty }(w)\sim w^{-(1+\gamma_{)}},$$

where γ≥1 is the Pareto index. This finding is called Pareto’s Law. In general, when γ→1, the distribution of social wealth is extremely unequal. Slanina [14] draws an analogy with inelastic granular gases to propose a model for social wealth exchange, where the dynamics is governed by a kinetic equation admitting self-similar solutions with a power-law tail, and analytically derives a closed-form wealth distribution under the continuous trading limit. Cordier *et al*. [15] present the exchange rule, discuss the effect of collision among agents on wealth distribution, and demonstrate that the steady-state wealth distribution has a Pareto tail. In the interaction rule, the saving propensity could affect the wealth distribution of agents. The authors in [16] study the situation where the two interacting parties have the same saving propensity, while Chatterjee *et al*. [17] suppose that the saving propensities of the two agents are different. The steady-state wealth distributions derived in [16,17] follow the power-law distribution. Düring and Toscani [18] revise the saving parameters in the interaction rule and find that when there is a significant difference in the saving tendencies of the two groups of agents, the distribution of the total wealth presents a bimodal feature. Bertotti [19] investigates how taxation and redistribution affect the distribution of wealth in a discrete framework, while Bisi *et al*. [20] examine the same issue within a continuous framework. The investigations in [19,20] demonstrate that taxation and redistribution could reduce wealth inequality. Pareschi and Toscani [21] explore the impact of knowledge on the wealth distribution and explain that the knowledge would promote the emergence of the elite class and exacerbate the wealth inequality. Supposing that there are three groups of agents including the susceptible, the infected and the recovered, Dimarco *et al*. [22] analyze the impact of disease transmission on wealth distribution and infer that diseases lead to a reduction in the size of the middle class and expand the wealth gap among agents. From the perspective of epidemic dynamics, Zhang *et al*. [23] demonstrate that the wealth densities of both the susceptible and the infected populations conform to a unimodal inverse gamma distribution. Bernardi *et al*. [24] examine the impact of large-scale vaccination campaigns on wealth distribution, and find that these vaccination efforts significantly contribute to reducing wealth disparities among agents. Adding a control term to the dynamic model, Wang *et al*. [25] achieve a result that the imbalance of decision-making capacity among agents would deteriorate the wealth inequality. Chen *et al*. [26] investigate the influence of leader’s abilities on decision-making consensus and conclude that the leader with high risk tolerance and control capacity could promote the team to reach consensus. Assuming that there are two types of goods in the market, Sun and Wang [27] categorize agents into two groups based on their differing trading propensities for each goods, allow agents to transfer between the groups, and find that agents’ transitions improve the wealth inequality. Bisi [28] investigates the wealth exchange between agents of two groups and allows agents to change their groups.

In the kinetic model of wealth distribution, the interaction among agents not only depends on the interaction rule, but also involves the collision kernel. The collision kernel describes the interaction frequency between agents and is divided into two types: Maxwellian collision kernels (constant collision kernels) and non-Maxwellian collision kernels. The former indicates that the interaction frequency among agents is independent of agents’ wealth, while the latter is the opposite. Toscani [29] establishes a dynamic model containing a constant collision kernel to illustrate the process of opinion formation, indicating that opinion exchange and information diffusion affect the opinion distribution of agents. Utilizing a Maxwellian collision kernel, Albi *et al*. [30] discuss the role of opinion leaders in opinion formation, arguing that the opinions of leaders have a guiding effect on followers. Zhong *et al*. [31] give the interaction rule containing a value function to measure the investment choice behavior of agents and build the Boltzmann equation with a constant collision kernel. The conclusion in [31] shows that the steady-state wealth distribution approaches the lognormal distribution. In the case where the interaction frequency relies on wealth, Furioli *et al*. [32] demonstrate that the wealth distribution of agents in a multi-agent system converges exponentially to its steady state. Under the premise that interaction frequency is a linear function of agents’ wealth, Zhou *et al*. [33] examine how the wealth substitution rate between agents affects wealth distribution, finding that high wealth substitution rates lead to a equitable wealth distribution. Meng *et al*. [34] construct a dynamic model containing a non-Maxwellian collision kernel, embed the tax and redistribution operators in the model, and state that tax and redistribution are conducive to wealth equality. Assuming that the student’s interaction frequency is determined by their GPA ( the grade point average), Hu and Chen [35] examine the effect of student interaction on GPA and find out the reversing conditions of GPA. Wang and Lai [36] develop a kinetic model to characterize wealth distribution in the financial market, and incorporate a wealth-dependent collision kernel to investigate the impact of varying trading frequency on wealth distribution. If trading frequency and propensity depend on the wealth of agents, Liu *et al*. [37] demonstrate that if trading propensity increases with wealth, the rich invest a high fraction of their capital in a single transaction, leading to a reduction in the agent’s wealth disparity.

To sum up, among the core references of this paper, Zhou *et al*. [33] and Boghosian *et al*. [38] do not consider the transfer behaviors of agents. Bisi [28] and Zhou *et al*. [33] assume that the expectation of random variables in the interaction rules is 0, with no consideration given to the case where the expectation of random variable relies on wealth. Zhang *et al*. [23], Bisi [28] and Boghosian *et al*. [38] utilize the Maxwellian collision kernel in their kinetic models. Based on the above summary, this paper employs the method of statistical physics to investigate the wealth exchange between agents of two groups and allows agents to transfer between the two groups. The specific differences are reflected in the following aspects.

(i) In the interaction rule proposed by Bisi [28], the mathematical expectation of the random variable is 0, implying that market uncertainty does not impact trading in a mean sense. Boghosian *et al*. [38] assume that the mathematical expectations of random variables are related to the wealth gap between the two interacting agents (see (5)). In this work, we still utilize the same assumption for random variables as that in [38].

(ii) Bisi [28] adopts a Maxwellian collision kernel, where the interaction frequency of agents is fixed. In this work, we adopt the non-Maxwellian collision kernel used in Zhou *et al*. [33] to represent the interaction frequency (see (7)).

(iii) Assuming that only agents from group 1 remain in the market when t→∞, Bisi [28] derives the steady-state wealth distribution of these agents. Inspired by the work in [23], we add an alternative case where the steady-state wealth distributions of agents in two groups maintain a fixed ratio ($g_{2,\infty }(w)=\lambda g_{1,\infty }(w)$, *λ* is a positive constant), and obtain the steady-state distributions for two groups of agents as well as the total wealth distribution of the market.

This paper proceeds as follows. In section 1, we develop a kinetic model to describe the interaction between agents from two groups. In section 2, we present the interaction rule and construct the interaction and transition operators. Section 3 aims at performing a Fokker–Planck transformation on the Boltzmann equation. In section 4, we find the steady-state solutions for two special cases and discuss the effects of parameters on the wealth distribution through numerical experiments. The conclusions are summarized in section 5.

## 1 The kinetic description for wealth exchange and individual transfers

We assume that the participants of wealth exchange are agents from two groups (groups 1 and 2). An agent could choose to transact with another agent in his own group or a separate group, and the agent could also choose to change his group after a single transaction. In this paper, the change of agent’s group is called agent transfer. In order to discuss the evolutions of wealth distributions of agents in two groups, in our model, interactions between agents follow certain rules, and the impacts of interactions and migrations of agents on wealth distribution are characterized by an interaction operator and a transfer operator, respectively.

The wealth densities of agents in groups 1 and 2 are represented by *f*_*i*_(*w*,*t*) (i=1,2), respectively. The wealth distribution *f*_*i*_(*w*,*t*) is uniquely characterized by the agent’s wealth *w* > 0 (*w* is dimensionless) and time *t* > 0. In general, there is no debt allowed in the wealth exchange model, that is, the agent with negative wealth does not participate in the interaction. When *w* = 0, the boundary condition *f*_*i*_(0,*t*) = 0 is given. The aggregate wealth distribution *f*(*w*,*t*) is a combination of the wealth distributions of agents from both groups, that is,

$$f(w,t)=f_{1}(w,t)+f_{2}(w,t).$$ (2)

Since *f*(*w*, *t*) is a probability density function, it satisfies the property that


$$\int_{R_{+}}f(w,t)dw=1,\;t>0.$$

The wealth exchanges and transfer behaviors of agents cause the wealth distribution to change over time. The evolution equation of the wealth distribution *f*_*i*_(*w*,*t*) becomes

$$\frac{\partial f_{i}(w,t)}{\partial t}=\sum_{j=1}^{2}Q_{i}(f_{i},f_{j})(w)+Q_{i}^{T}(f_{1},f_{2})(w),\;i=1,2.$$ (3)

In (3), $Q_{i}(f_{i},f_{j})(w)$ is the binary interaction operator, which measures the effect of the transaction on wealth distribution. The evolution of wealth distribution under agents’ migration is governed by the operator $Q_{i}^{T}(f_{1},f_{2})(w)$.

**Fig 1:**
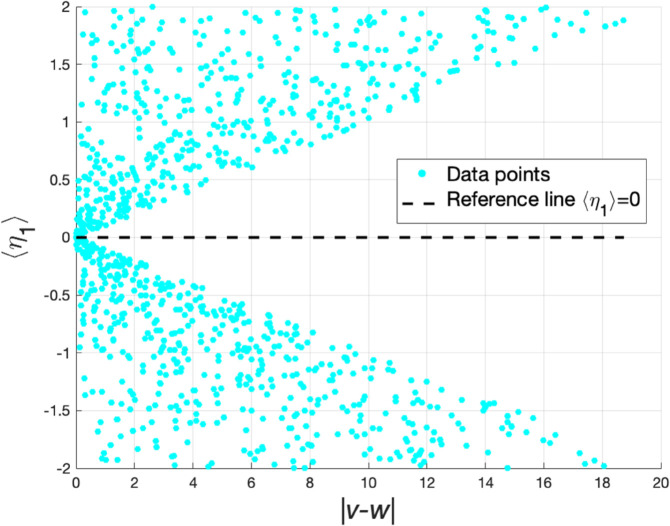
The relationship between |v−w| and $\langle \eta_{1}\rangle$.

Next, we describe the process of wealth exchange of agents. We use (*w*,*v*) and (*w* ,*v* ) to represent the pre-transaction and the post-transaction wealths of agents in a transaction, respectively. The trading rule is written as

$$\left\{ \begin{array}{c} w^{*}=w+\theta (v-w)+\eta_{1}w,\;v^{*}=v+\theta (w-v)+\eta_{2}v, \end{array}\right.$$ (4)

where *θ* represents the trading rate of the agent, $\eta_{1}$ and $\eta_{2}$ are independent random variables. Different from the assumption that the expectation of random variable is 0 in [28], Boghosian *et al*. [38] suppose that the expectations of random variables are related to the wealth gap between the two sides of the interaction, which are expressed in the following forms

$$\langle \eta_{1}\rangle =\xi \frac{v-w}{w},\;\langle \eta_{2}\rangle =\xi \frac{w-v}{v},$$ (5)

where ⟨·⟩ denotes the mathematical expectation and 0<ξ<1 is a proportional constant. Besides, the expectations of the square of random variables satisfy $\langle \eta_{1}^{2}\rangle =\langle \eta_{2}^{2}\rangle =\sigma$. Taking $\eta_{1}$ as an example, in Fig 1, we randomly generate 1000 points and display the relationship between the wealth gap |v−w| among agents and the expectation of the random variable $\eta_{1}$. It is seen that when the wealth gap among agents increases, the degree of deviation between the data points and the reference line $\langle \eta_{1}\rangle =0$ increases. This phenomenon indicates that the transaction risk for two agents with an excessive wealth gap is higher than that for two agents with comparable wealth. The result of $\eta_{2}$ is similar.

Since there are random variables in trading rule (4), the binary interaction operator should be written in the form of mathematics expectation

$$\int_{R_{+}}\varphi (w)Q_{i}(f_{i},f_{j})(w)dw=\left\langle \int_{R_{+}^{2}}K_{i}(w,v)[\varphi (w^{*})-\varphi (w)]f_{i}(w)f_{j}(v)dwdv\right\rangle ,$$ (6)

where φ(w) is a smooth function with supported set in $R_{+}^{2}$. According to the ideas in [33], for i=1,2, we also assume that the corresponding forms of collision kernels are

$$K_{1}(w,v)=\alpha w+\beta v,\;K_{2}(w,v)=\beta w+\alpha v,$$ (7)

where *α* and *β* indicate the proportions of assets put into the market by agents in groups 1 and 2, respectively. Referring to the properties of the linear utility function, Zhou *et al*. [33] call α/β (or β/α) as the wealth replacement rate. α/β represents that if the agent with wealth *w* invests an additional 1 unit of wealth, the wealth contribution of agent with wealth *v* decreases by α/β units. Similarly, β/α denotes that a unit increase in wealth exchange by the agent with wealth *v* corresponds to a reduction of β/α units in wealth investment by the agent with wealth *w*.

We consider a transfer process in which one of the agents in an interaction chooses to change his group, including the following four transfer types.


(a)1+1→1+2,(b)2+2→1+2,(c)1+2→1+1,(d)1+2→2+2.

The transfer (*a*) refers to a transaction between two agents from group 1, after which one of the agents immigrates to group 2. The explanations for transfers (b), (c) and (d) are similar. Supposing that the probability of an agent from group 1 immigrating to group 2 is *p*_12_ ($0\leq p_{12}\leq 1$), and the probability of an agent from group 2 immigrating to group 1 is *p*_21_ ($0\leq p_{21}\leq 1$). Using P(·) to denote the probability of the event in parentheses occurring, for the above four transfer types, we have *P*(*a*) = *P*(*d*) = *p*_12_ and *P*(*b*) = *P*(*c*) = *p*_21_. The total transfer operator is expressed as the sum of operators of transfers (*a*)–(*d*), that is,


$$Q_{i}^{T}(f_{1},f_{2})(w)=\sum_{l\in \{a,b,c,d\}}Q_{i}^{T(l)}(f_{1},f_{2})(w),\;i=1,2.$$

Taking transfer (*a*) as an example, we provide a detailed explanation of the construction process of the transfer operator. We denote the wealth of the two agents before the transition as $\left(w_{*},v_{*}\right)$, and the wealth after the transition as (*w*,*v*). *f*(*w*,*t*)*dw* denotes the number of agents whose wealth falls within the interval (w,w  +  dw). Thus, we use $p_{12}f_{1}(w_{*},t)f_{1}(v_{*},t)dw_{*}dv_{*}$ (*p*_12_ is the probability of agents transferring from group 1 to group 2) to represent the number of agents whose wealth changes from $\left(w_{*},v_{*}\right)$ to (*w*,*v*) after the transition. We refer to $p_{12}f_{1}(w_{*},t)f_{1}(v_{*},t)dw_{*}dv_{*}$ as the gain term for agents with wealth (*w*,*v*). Similarly, $p_{12}f_{1}(w,t)f_{1}(v,t)dwdv$ denotes the number of agents whose wealth is no longer (*w*,*v*) after the transition. Thus, $p_{12}f_{1}(w,t)f_{1}(v,t)dwdv$ serves as the loss term for agents with wealth (*w*,*v*). In transfer (*a*), there is a net decrease of one unit in the number of agents belonging to group 1. Thus, we have


$$Q_{1}^{T(a)}(w)dw=p_{12}f_{1}(w_{*},t)f_{1}(v_{*},t)dw_{*}dv_{*}-2p_{12}f_{1}(w,t)f_{1}(v,t)dwdv.$$

For agents in group 2, there is a net increase of one unit, that is,


$$Q_{2}^{T(a)}(w)dw=p_{12}f_{1}(w_{*},t)f_{1}(v_{*},t)dw_{*}dv_{*}.$$

We express operators $Q_{1}^{T(a)}$ and $Q_{2}^{T(a)}$ in their weak forms and obtain


$$\int_{R_{+}}\varphi (w)Q_{1}^{T(a)}(w)dw\;=\;p_{12}\int_{R_{+}^{2}}\varphi (w)f_{1}(w_{*},t)f_{1}(v_{*},t)dw_{*}dv_{*}\;-2p_{12}\int_{R_{+}^{2}}\varphi (w)f_{1}(w,t)f_{1}(v,t)dwdv$$

and


$$\int_{R_{+}}\varphi (w)Q_{2}^{T(a)}(w)dw=p_{12}\int_{R_{+}^{2}}\varphi (w)f_{1}(w_{*},t)f_{1}(v_{*},t)dw_{*}dv_{*}.$$

The construction processes of operators of transfers (*b*) – (*d*) are referred to [28], we write the transfer operators in their weak forms


$$\int_{R_{+}}\varphi (w)Q_{1}^{T(b)}(w)dw=p_{21}\int_{R_{+}^{2}}\varphi (w)f_{2}(w_{*})f_{2}(v_{*})dw_{*}dv_{*},$$

$$\int_{R_{+}}\varphi (w)Q_{2}^{T(b)}(w)dw\;=\;p_{21}\int_{R_{+}^{2}}\varphi (w)f_{2}(w_{*})f_{2}(v_{*})dw_{*}dv_{*}\;-2p_{21}\int_{R_{+}^{2}}\varphi (w)f_{2}(w)f_{2}(v)dwdv,$$

$$\int_{R_{+}}\varphi (w)Q_{1}^{T(c)}(w)dw\;=\;p_{21}\int_{R_{+}^{2}}\varphi (w)f_{1}(w_{*})f_{2}(v_{*})dw_{*}dv_{*}\;+p_{21}\int_{R_{+}^{2}}\varphi (w)f_{1}({\overset{―}{w}}_{*})f_{2}({\overset{―}{v}}_{*})d{\overset{―}{w}}_{*}d{\overset{―}{v}}_{*}\;-p_{21}\int_{R_{+}^{2}}\varphi (w)f_{1}(w)f_{2}(v)dwdv,$$

$$\int_{R_{+}}\varphi (w)Q_{2}^{T(c)}(w)dw=p_{21}\int_{R_{+}^{2}}\varphi (w)f_{2}(w)f_{1}(v)dwdv,$$

$$\int_{R_{+}}\varphi (w)Q_{1}^{T(d)}(w)dw=-p_{12}\int_{R_{+}^{2}}\varphi (w)f_{1}(w)f_{2}(v)dwdv,$$

$$\int_{R_{+}}\varphi (w)Q_{2}^{T(d)}(w)dw\;=\;p_{12}\int_{R_{+}^{2}}\varphi (w)f_{1}(w_{*})f_{2}(v_{*})dw_{*}dv_{*}\;+p_{12}\int_{R_{+}^{2}}\varphi (w)f_{1}({\overset{―}{w}}_{*})f_{2}({\overset{―}{v}}_{*})d{\overset{―}{w}}_{*}d{\overset{―}{v}}_{*}\;-p_{12}\int_{R_{+}^{2}}\varphi (w)f_{1}(v)f_{2}(w)dwdv,$$

where $\left({\overset{―}{w}}_{*},{\overset{―}{v}}_{*}\right)$ represents the wealth of agents before the transition, and the wealth of agents after the transition is (*w*,*v*). In transfers (*c*) and (*d*), the two parties before the transition are from different groups. Thus, swapping their positions in the derivation is not allowed. That is, although the wealth of both parties after the transition are (*w*,*v*), the wealth before the transition could be $\left(w_{*},v_{*}\right)$ or $\left({\overset{―}{w}}_{*},{\overset{―}{v}}_{*}\right)$, and $\left(w_{*},v_{*})\neq ({\overset{―}{w}}_{*},{\overset{―}{v}}_{*}\right)$.

We write the Boltzmann Eq (3) into its weak form, that is,

$$\;\frac{d}{dt}\int_{R_{+}}\varphi (w)f_{1}(w,t)dw\;=\int_{R_{+}^{2}}K_{1}(w,v)\left\langle \varphi (w^{*})-\varphi (w)\right\rangle f_{1}(w)f_{1}(v)dwdv\;\;+\int_{R_{+}^{2}}K_{1}(w,v)\left\langle \varphi (w^{*})-\varphi (w)\right\rangle f_{1}(w)f_{2}(v)dwdv\;\;+p_{12}\int_{R_{+}^{2}}\varphi (w)f_{1}(w_{*})f_{1}(v_{*})dw_{*}dv_{*}-2p_{12}\int_{R_{+}^{2}}\varphi (w)f_{1}(w)f_{1}(v)dwdv\;\;+p_{21}\int_{R_{+}^{2}}\varphi (w)f_{2}(w_{*})f_{2}(v_{*})dw_{*}dv_{*}+p_{21}\int_{R_{+}^{2}}\varphi (w)f_{1}(w_{*})f_{2}(v_{*})dw_{*}dv_{*}\;\;+p_{21}\int_{R_{+}^{2}}\varphi (w)f_{1}({\overset{―}{w}}_{*})f_{2}({\overset{―}{v}}_{*})d{\overset{―}{w}}_{*}d{\overset{―}{v}}_{*}-p_{21}\int_{R_{+}^{2}}\varphi (w)f_{1}(w)f_{2}(v)dwdv\;\;-p_{12}\int_{R_{+}^{2}}\varphi (w)f_{1}(w)f_{2}(v)dwdv$$ (8)

and

$$\;\frac{d}{dt}\int_{R_{+}}\varphi (w)f_{2}(w,t)dw\;=\int_{R_{+}^{2}}K_{2}(w,v)\left\langle \varphi (w^{*})\varphi (w)\right\rangle f_{2}(w)f_{2}(v)dwdv\;\;+\int_{R_{+}^{2}}K_{2}(w,v)\left\langle \varphi (w^{*})-\varphi (w)\right\rangle f_{2}(w)f_{1}(v)dwdv\;\;+p_{12}\int_{R_{+}^{2}}\varphi (w)f_{1}(w_{*})f_{1}(v_{*})dw_{*}dv_{*}+p_{21}\int_{R_{+}^{2}}\varphi (w)f_{2}(w_{*})f_{2}(v_{*})dw_{*}dv_{*}\;\;-2p_{21}\int_{R_{+}^{2}}\varphi (w)f_{2}(w)f_{2}(v)dwdv+p_{21}\int_{R_{+}^{2}}\varphi (w)f_{2}(w)f_{1}(v)dwdv\;\;+p_{12}\int_{R_{+}^{2}}\varphi (w)f_{1}(w_{*})f_{2}(v_{*})dw_{*}dv_{*}+p_{12}\int_{R_{+}^{2}}\varphi (w)f_{1}({\overset{―}{w}}_{*})f_{2}({\overset{―}{v}}_{*})d{\overset{―}{w}}_{*}d{\overset{―}{v}}_{*}\;\;-p_{12}\int_{R_{+}^{2}}\varphi (w)f_{1}(v)f_{2}(w)dwdv.$$ (9)

## 2 Properties of moments of the wealth distribution

In the dynamic model presented in the previous section, binary interactions lead to the flow of wealth between the two groups, and individuals’ transfer behaviors cause the movement of the population between the two groups. This section aims to derive the evolution equations for the number of people and the mean wealth in each group, and prove that the first-order moment of the total wealth distribution function of the system is conserved.

Define the moments of wealth distribution *f*_*i*_(*w*,*t*) as


$$m_{i,s}(t)=\int_{R_{+}}w^{s}f_{i}(w,t)dw,\;s\geq 0,$$

where *s* represents the order of moments. When *s* = 0, we have


$$\rho_{1}(t)=\int_{R_{+}}f_{1}(w,t)dw,\;\rho_{2}(t)=\int_{R_{+}}f_{2}(w,t)dw.$$

$\rho_{1}(t)$ and $\rho_{2}(t)$ vary with the agents’ transfer and denote the proportions of the number of agents in groups 1 and 2 to the total number of agents in the market, respectively. At any time *t* > 0, $\rho_{1}(t)$ and $\rho_{2}(t)$ satisfy

$$\rho_{1}(t)+\rho_{2}(t)=1.$$ (10)

By choosing φ(w)=1 in Eqs (8) and (9), we obtain

$$\left\{ \begin{array}{c} \frac{d\rho_{1}(t)}{dt}=p_{21}\rho_{2}^{2}(t)-p_{12}\rho_{1}^{2}(t)+p_{21}\rho_{1}(t)\rho_{2}(t)-p_{12}\rho_{1}(t)\rho_{2}(t)\\ \frac{d\rho_{2}(t)}{dt}=-p_{21}\rho_{2}^{2}(t)+p_{12}\rho_{1}^{2}(t)-p_{21}\rho_{1}(t)\rho_{2}(t)+p_{12}\rho_{1}(t)\rho_{2}(t). \end{array}\right.$$ (11)

When the following condition is satisfied, system (11) reach a steady state.

$$\rho_{2}=k\rho_{1},\;k=\frac{-(p_{21}-p_{12})+\sqrt{(p_{21}-p_{12}{)}^{2}+4p_{12}p_{21}}}{2p_{21}}.$$ (12)

Therefore, combining with Eq (10), the proportions of the number of agents in the two groups to the total number of agents in the steady state are

$$(\rho_{1}{)}_{\infty }=\frac{1}{1+k},\;(\rho_{2}{)}_{\infty }=\frac{k}{1+k}.$$ (13)

When *s* = 1, *m*_1,1_(*t*) and *m*_2,1_(*t*) indicate the mean wealth of agents from groups 1 and 2, which are written as


$$m_{1,1}(t)=\int_{R_{+}}wf_{1}(w,t)dw,\;m_{2,1}(t)=\int_{R_{+}}wf_{2}(w,t)dw.$$

According to the exchange rule (4), we have


$$\langle w^{*}+v^{*}\rangle =w+v+\langle \eta_{1}\rangle w+\langle \eta_{2}\rangle v=w+v.$$

By setting φ(w)=w, Eq (6) becomes


$$\int_{R_{+}}wQ_{1}(f_{1},f_{2})(w)dw=\frac{1}{2}\int_{R_{+}^{2}}K_{1}(w,v)\left\langle w^{*}+v^{*}-w-v\right\rangle f_{1}(w)f_{1}(v)dwdv\;\;+\frac{1}{2}\int_{R_{+}^{2}}K_{1}(w,v)\left\langle w^{*}+v^{*}-w-v\right\rangle f_{1}(w)f_{2}(v)dwdv\;=0.$$

Similarly, we obtain $\int_{R_{+}}wQ_{2}(f_{1},f_{2})(w)dw=0$. In other words, binary interactions do not affect the evolution of the first-order moment of the wealth distribution function. Next, we consider whether individual transfers affect the evolution of the first-order moment. Substituting φ(w)=w into the weak form of the transfer operator yields


$$\int_{R_{+}}wQ_{1}^{T}(f_{1},f_{2})(w)dw=-p_{12}\rho_{1}m_{1,1}(t)+p_{21}\rho_{2}m_{2,1}(t)+p_{21}\rho_{1}m_{2,1}(t)-p_{12}\rho_{2}m_{1,1}(t)$$

and


$$\int_{R_{+}}wQ_{2}^{T}(f_{1},f_{2})(w)dw=p_{12}\rho_{1}m_{1,1}(t)-p_{21}\rho_{2}m_{2,1}(t)-p_{21}\rho_{1}m_{2,1}(t)+p_{12}\rho_{2}m_{1,1}(t).$$

Overall, the evolution equation for the first-order moment of the total wealth distribution function is


$$\frac{d}{dt}\int_{R_{+}}wf(w,t)dw=\frac{d}{dt}\int_{R_{+}}wf_{1}(w,t)dw+\frac{d}{dt}\int_{R_{+}}wf_{2}(w,t)dw\;=\int_{R_{+}}wQ_{1}(f_{1},f_{2})(w)dw+\int_{R_{+}}wQ_{1}^{T}(f_{1},f_{2})(w)dw\;\;+\int_{R_{+}}wQ_{2}(f_{1},f_{2})(w)dw+\int_{R_{+}}wQ_{2}^{T}(f_{1},f_{2})(w)dw\;=0.$$

This implies that the first-order moment of the total wealth distribution does not vary with time. On this basis, it is reasonable to assume that

$$(m_{1,1}{)}_{\infty }+(m_{2,1}{)}_{\infty }=\int_{R_{+}}wf_{\infty }(w)dw={\bar{m}}_{1},$$ (14)

where ${\bar{m}}_{1}$ is a positive constant.

Due to the complexity of the non-Maxwellian collision kernel adopted in this paper, we assume that the sum of the second moments of *f*_1_(*w*,*t*) and *f*_2_(*w*,*t*) (the second moment of the total wealth distribution) is a constant when t→∞. Defining $m_{1,2}(t)=\int_{R_{+}}w^{2}f_{1}(w,t)dw$ and $m_{2,2}(t)=\int_{R_{+}}w^{2}f_{2}(w,t)dw$, we have

$$(m_{1,2}{)}_{\infty }+(m_{2,2}{)}_{\infty }=\int_{R_{+}}w^{2}f_{\infty }(w)dw={\bar{m}}_{2},$$ (15)

where ${\bar{m}}_{2}$ is a positive constant, and ${\bar{m}}_{2}$ is treated as an exogenous constraint that determines the steady-state distribution function.

Main notations introduced in this paper are summarized in the Table 1.

**Table 1:** Summary of key model parameters.

Parameter symbol	Parameter meaning	Range of values
*α*	The proportion of wealth invested by the agent with wealth *w*.	0<α≤1
*β*	The proportion of wealth invested by the agent with wealth *v*.	0<β≤1
*θ*	The trading rate of agents.	0≤θ≤1
ξ	The proportional parameter of the expectation of random variables.	0<ξ<1
*σ*	The expectation of the square of a random variable.	0<σ<+∞
*p*_12_	The probability of an agent transferring from group 1 to group 2.	$0\leq p_{12}\leq 1$
*p*_21_	The probability of an agent transferring from group 2 to group 1.	$0\leq p_{21}\leq 1$
*λ*	The ratio of the steady-state wealth distributions of the two groups of agents.	0<λ<+∞
$\rho_{i}$	The proportion of the number of agents in group *i* to the total number of agents.	$0\leq \rho_{i}\leq 1$
*m*_*i*,*s*_	The s-th moment of the wealth distribution of agents in group *i*.	$0<m_{i,s}<+\infty$

## 3 Derivation of the Fokker-Planck equation

In fact, it is difficult to solve the Boltzmann Eqs (8) and (9) (see [28]). Through the approach of continuous trading limits, we convert the Boltzmann equations into the Fokker-Planck equations and calculate its steady-state solutions under certain conditions. A constant 0<ϵ≪1 is used to scale the parameters, the entire market is regarded as a continuous market composing of infinite tiny transactions. The scaled parameters are as follows.


$$\theta \to \epsilon \theta ,\;p_{12}\to \epsilon p_{12},\;p_{21}\to \epsilon p_{21},\;\sigma \to \epsilon \sigma ,\;\xi \to \epsilon \xi .$$

After scaling, there are $\langle \eta_{1}\rangle \to \epsilon \xi \frac{v-w}{w}$, $\langle \eta_{2}\rangle \to \epsilon \xi \frac{w-v}{v}$ and $\langle \eta_{i}^{2}\rangle \to \epsilon \sigma$ (i=1,2). Performing the Taylor expansion of the smooth function $\varphi (w^{*})$, we obtain


$$\varphi (w^{*})-\varphi (w)=\varphi^{\prime }(w)(w^{*}-w)+\frac{\varphi^{\prime \prime }(w)}{2}(w^{*}-w{)}^{2}+\frac{\varphi^{\prime \prime \prime }(\tilde{w})}{6}(w^{*}-w{)}^{3},$$

where $\tilde{w}=\kappa w^{*}+(1-\kappa )w$ (0≤κ≤1). According to the exchange rule (4), we have


$$\langle w^{*}-w\rangle =\epsilon (\theta +\xi )(v-w)$$

and


$$\langle (w^{*}-w{)}^{2}\rangle =\epsilon^{2}(\theta^{2}+2\theta \xi )(v-w{)}^{2}+\epsilon \sigma w^{2}.$$

The expectation of the Taylor expansion of $\varphi (w^{*})$ is

$$\langle \varphi (w^{*})-\varphi (w)\rangle =\epsilon (\theta +\xi )\varphi^{{\;}^{\prime }}(w)(v-w)+\frac{\epsilon \sigma }{2}\varphi^{{\;}^{\prime \prime }}(w)w^{2}+R_{\epsilon }(w,v),$$ (16)

where $R_{\epsilon }(w,v)=\frac{\epsilon^{2}(\theta^{2}+2\theta \xi )}{2}\varphi^{\prime \prime }(w)(v-w{)}^{2}+<\frac{\varphi^{\prime \prime \prime }(\tilde{w})}{6}(w^{*}-w{)}^{3}>$. In (8), the first two terms on the right side is written as

$$\;\int_{R_{+}^{2}}K_{1}(w,v)\left\langle \varphi (w^{*})-\varphi (w)\right\rangle f_{1}(w,t)f_{1}(v,t)dwdv\;\;+\int_{R_{+}^{2}}K_{1}(w,v)\left\langle \varphi (w^{*})-\varphi (w)\right\rangle f_{1}(w,t)f_{2}(v,t)dwdv\;=\epsilon \int_{R_{+}^{2}}(\alpha w+\beta v)\left[(\theta +\xi )\varphi^{{\;}^{\prime }}(w)(v-w)+\frac{\sigma }{2}\varphi^{{\;}^{\prime \prime }}(w)w^{2}\right]f_{1}(w,t)f_{1}(v,t)dwdv\;\;+\epsilon \int_{R_{+}^{2}}(\alpha w+\beta v)\left[(\theta +\xi )\varphi^{{\;}^{\prime }}(w)(v-w)+\frac{\sigma }{2}\varphi^{{\;}^{\prime \prime }}(w)w^{2}\right]f_{1}(w,t)f_{2}(v,t)dwdv\;\;+{\tilde{R}}_{\epsilon }(w,v),$$ (17)

where


$${\tilde{R}}_{\epsilon }(w,v)\;=\;\int_{R_{+}^{2}}(\alpha w+\beta v)R_{\epsilon }(w,v)f_{1}(w,t)f_{1}(v,t)dwdv\;+\int_{R_{+}^{2}}(\alpha w+\beta v)R_{\epsilon }(w,v)f_{1}(w,t)f_{2}(v,t)dwdv.$$

When ϵ→0, ${\tilde{R}}_{\epsilon }(w,v)=0$. After scaling τ=ϵt, we set $g_{i}(w,\tau )=f_{i}(w,t)$ (i=1,2). Using integration by parts, from (17), we derive that

$$\;\int_{R_{+}^{2}}K_{1}(w,v)\langle \varphi (w^{*})-\varphi (w)\rangle g_{1}(w,\tau )g_{1}(v,\tau )dwdv\;\;+\int_{R_{+}^{2}}K_{1}(w,v)\langle \varphi (w^{*})-\varphi (w)\rangle g_{1}(w,\tau )g_{2}(v,\tau )dwdv\;=\epsilon (\theta +\xi )[(\beta -\alpha )(m_{1,1}(\tau )+m_{2,1}(\tau ))\int_{R_{+}}\varphi (w)\frac{\partial }{\partial w}(wg_{1}(w,\tau ))dw\;\;+\alpha (\rho_{1}(\tau )+\rho_{2}(\tau ))\int_{R_{+}}\varphi (w)\frac{\partial }{\partial w}(w^{2}g_{1}(w,\tau ))dw\;\;-\beta (m_{1,2}(\tau )+m_{2,2}(\tau ))\int_{R_{+}}\varphi (w)\frac{\partial }{\partial w}g_{1}(w,\tau )dw]\;\;+\frac{\epsilon \sigma }{2}[\alpha (\rho_{1}(\tau )+\rho_{2}(\tau ))\int_{R_{+}}\varphi (w)\frac{\partial^{2}}{\partial w^{2}}(w^{3}g_{1}(w,\tau ))dw\;\;+\beta (m_{1,1}(\tau )+m_{2,1}(\tau ))\int_{R_{+}}\varphi (w)\frac{\partial^{2}}{\partial w^{2}}(w^{2}g_{1}(w,\tau ))dw].$$ (18)

Substituting (18) into (8), we derive the Fokker-Planck equation for the wealth density of the agent in group 1.

$$\frac{\partial g_{1}(w,\tau )}{\partial \tau }\;=\;(\theta +\xi )(\beta -\alpha )(m_{1,1}(\tau )+m_{2,1}(\tau ))\frac{\partial }{\partial w}(wg_{1}(w,\tau ))\;+\alpha (\theta +\xi )(\rho_{1}(\tau )+\rho_{2}(\tau ))\frac{\partial }{\partial w}(w^{2}g_{1}(w,\tau ))\;-\beta (\theta +\xi )(m_{1,2}(\tau )+m_{2,2}(\tau ))\frac{\partial }{\partial w}g_{1}(w,\tau )\;+\frac{\sigma \alpha (\rho_{1}(\tau )+\rho_{2}(\tau ))}{2}\frac{\partial^{2}}{\partial w^{2}}(w^{3}g_{1}(w,\tau ))\;+\frac{\sigma \beta (m_{1,1}(\tau )+m_{2,1}(\tau ))}{2}\frac{\partial^{2}}{\partial w^{2}}(w^{2}g_{1}(w,\tau ))\;+p_{21}g_{2}(w,\tau )-p_{12}g_{1}(w,\tau ).$$ (19)

Utilizing the same method, the Fokker-Planck equation for $g_{2}(w,\tau )$ is expressed as

$$\frac{\partial g_{2}(w,\tau )}{\partial \tau }\;=\;(\theta +\xi )(\alpha -\beta )(m_{1,1}(\tau )+m_{2,1}(\tau ))\frac{\partial }{\partial w}(wg_{2}(w,\tau ))\;+\beta (\theta +\xi )(\rho_{1}(\tau )+\rho_{2}(\tau ))\frac{\partial }{\partial w}(w^{2}g_{2}(w,\tau ))\;-\alpha (\theta +\xi )(m_{1,2}(\tau )+m_{2,2}(\tau ))\frac{\partial }{\partial w}g_{2}(w,\tau )\;+\frac{\sigma \beta (\rho_{1}(\tau )+\rho_{2}(\tau ))}{2}\frac{\partial^{2}}{\partial w^{2}}(w^{3}g_{2}(w,\tau ))\;+\frac{\sigma \alpha (m_{1,1}(\tau )+m_{2,1}(\tau ))}{2}\frac{\partial^{2}}{\partial w^{2}}(w^{2}g_{2}(w,\tau ))\;-p_{21}g_{2}(w,\tau )+p_{12}g_{1}(w,\tau ).$$ (20)

## 4 The steady-state wealth distribution and numerical experiments

Combining assumptions (10)–(15), when τ→∞, we acquire the equilibrium states of Eqs (19) and (20) in the forms

$$\frac{\sigma }{2}\frac{\partial^{2}}{\partial w^{2}}[(\alpha w+\beta {\bar{m}}_{1})w^{2}g_{1,\infty }(w)]+(\theta +\xi )\frac{\partial }{\partial w}[(\alpha w^{2}+(\beta -\alpha ){\bar{m}}_{1}w\;-\beta {\bar{m}}_{2})g_{1,\infty }(w)]+p_{21}g_{2,\infty }(w)-p_{12}g_{1,\infty }(w)=0$$ (21)

and

$$\frac{\sigma }{2}\frac{\partial^{2}}{\partial w^{2}}[(\beta w+\alpha {\bar{m}}_{1})w^{2}g_{2,\infty }(w)]+(\theta +\xi )\frac{\partial }{\partial w}[(\beta w^{2}+(\alpha -\beta ){\bar{m}}_{1}w\;-\alpha {\bar{m}}_{2})g_{2,\infty }(w)]-p_{21}g_{2,\infty }(w)+p_{12}g_{1,\infty }(w)=0.$$ (22)

### 4.1 Solvable case 1

Considering a case where transition restrictions exist. Namely, we stipulate that only agents with extremely high wealth are eligible to transfer to group 2. In this situation, *p*_12_ becomes so small that satisfies $p_{12}=O(\varepsilon^{2})$ (*ε* is an extremely small constant, and *p*_12_ = 0 when ε→0). According to Eq (12), we know that *k* = 0 when *p*_12_ = 0. Thus, we obtain


$$(\rho_{1}{)}_{\infty }=\frac{1}{1+k}=1,\;(\rho_{2}{)}_{\infty }=\frac{k}{1+k}=0.$$

Correspondingly, according to conditions (10)–(15), there are $(m_{1,1}{)}_{\infty }={\bar{m}}_{1}$, $(m_{1,2}{)}_{\infty }={\bar{m}}_{2}$, $(m_{2,1}{)}_{\infty }=0$ and $(m_{2,2}{)}_{\infty }=0$. Then, under the condition that $g_{2,\infty }(w)$ disappears, $g_{1,\infty }(w)$ obeys the equation

$$(\theta +\xi )\frac{\partial }{\partial w}[(\alpha w^{2}+(\beta -\alpha ){\bar{m}}_{1}w-\beta {\bar{m}}_{2})g_{1,\infty }(w)]\;+\frac{\sigma }{2}\frac{\partial^{2}}{\partial w^{2}}[(\alpha w+\beta {\bar{m}}_{1})w^{2}g_{1,\infty }(w)]=0.$$ (23)

Solving (23), we obtain the steady-state solution

$$g_{1,\infty }(w)=C_{1}w^{A_{1}}(B_{1}w+B_{2}{)}^{A_{2}}exp\left\{-\frac{A_{3}}{w}\right\},$$ (24)

where *C*_1_ is a constant that meets the condition $\int_{R_{+}}g_{1,\infty }(w)dw=1$. In (24), constants *A*_1_, *A*_2_ and *A*_3_ are given by


$$A_{1}=\frac{2\left[(\theta +\xi )(\frac{\alpha }{\beta }-1)-\sigma \right]{\bar{m}}_{1}^{2}-2\frac{\alpha }{\beta }(\theta +\xi ){\bar{m}}_{2}}{\sigma {\bar{m}}_{1}^{2}},$$

$$A_{2}=\frac{2\frac{\alpha }{\beta }(\theta +\xi ){\bar{m}}_{2}-\left[2(\theta +\xi )(\frac{\alpha }{\beta }+2)+\sigma \right]{\bar{m}}_{1}^{2}}{\sigma {\bar{m}}_{1}^{2}},$$

$$A_{3}=\frac{2(\theta +\xi ){\bar{m}}_{2}}{\sigma {\bar{m}}_{1}^{2}},$$

and


$$B_{1}=\sigma \alpha ,\;B_{2}=\sigma \beta {\bar{m}}_{1}.$$

According to the ranges of the relevant parameters in Table 1, we know that *B*_1_>0 and *B*_2_>0. Therefore, for any *w*>0, the steady-state wealth distribution maintains positivity. Additionally, we maintain the normalization of $g_{1,\infty }(w)$ by solving for the constant *C*_1_ that makes the integral of $g_{1,\infty }(w)$ equal to 1. From the steady-state solution $g_{1,\infty }(w)$, we conclude that when α/β=1, the wealth replacement rate does not affect the wealth density $g_{1,\infty }(w)$. Conversely, when α/β≠1, the wealth density $g_{1,\infty }(w)$ is subject to the impact of wealth replacement rate. Furthermore, the wealth density $g_{1,\infty }(w)$ is also affected by the trading rate *θ* and market risk *σ*.

### 4.2 Solvable case 2

Referring to the perspective in [23], we discuss another situation in which the ratio of the wealth distributions of agents in the two groups remains stable as t→∞, that is, $g_{2,\infty }(w)=\lambda g_{1,\infty }(w)$ (*λ* is a positive constant). At this time, the proportions of the number of agents in groups 1 and 2 to the total number of agents depend on the transition probabilities *p*_12_ and *p*_21_, that is, $(\rho_{1}{)}_{\infty }$ and $(\rho_{2}{)}_{\infty }$ satisfy Eqs (12) and (13). $g_{1,\infty }(w)$ and $g_{2,\infty }(w)$ are solutions of following equations

$$\frac{\sigma }{2}\frac{\partial^{2}}{\partial w^{2}}[(\alpha w+\beta {\bar{m}}_{1})w^{2}g_{1,\infty }(w)]+(\theta +\xi )\frac{\partial }{\partial w}[(\alpha w^{2}+(\beta -\alpha ){\bar{m}}_{1}w\;-\beta {\bar{m}}_{2})g_{1,\infty }(w)]+(\lambda p_{21}-p_{12})g_{1,\infty }(w)=0,$$ (25)

$$\frac{\sigma }{2}\frac{\partial^{2}}{\partial w^{2}}[(\beta w+\alpha {\bar{m}}_{1})w^{2}g_{2,\infty }(w)]+(\theta +\xi )\frac{\partial }{\partial w}[(\beta w^{2}+(\alpha -\beta ){\bar{m}}_{1}w\;-\alpha {\bar{m}}_{2})g_{2,\infty }(w)]+\left(\frac{p_{12}}{\lambda }-p_{21}\right)g_{2,\infty }(w)=0.$$ (26)

From (25) and (26), we acquire

$$g_{1,\infty }(w)=C_{2}w^{A_{4}}(B_{1}w+B_{2}{)}^{A_{5}}exp\left\{-\frac{A_{6}}{w}\right\}$$ (27)

and

$$g_{2,\infty }(w)=C_{3}w^{A_{7}}(B_{3}w+B_{4}{)}^{A_{8}}exp\left\{-\frac{A_{9}}{w}\right\},$$ (28)

where constants *C*_2_ and *C*_3_ satisfy $\int_{R_{+}}g_{1,\infty }(w)dw+\int_{R_{+}}g_{2,\infty }(w)dw=1$. The constants in (27) and (28) are expressed as


$$A_{4}=\frac{2\left[(\theta +\xi )(\frac{\alpha }{\beta }-1)-\sigma \right]{\bar{m}}_{1}^{2}-2\frac{\alpha }{\beta }\left[(\theta +\xi ){\bar{m}}_{2}+\frac{p_{12}-\lambda p_{21}}{\beta }\right]}{\sigma {\bar{m}}_{1}^{2}},$$

$$A_{5}=\frac{2\frac{\alpha }{\beta }\left[(\theta +\xi ){\bar{m}}_{2}+\frac{p_{12}-\lambda p_{21}}{\beta }\right]-\left[2\frac{\alpha }{\beta }(\theta +\xi )-\sigma \right]{\bar{m}}_{1}^{2}}{\sigma {\bar{m}}_{1}^{2}},$$

$$A_{6}=\frac{2\left[(\theta +\xi ){\bar{m}}_{2}+\frac{p_{12}-\lambda p_{21}}{\beta }\right]}{\sigma {\bar{m}}_{1}},$$

$$A_{7}=\frac{2\left[(\theta +\xi )(\frac{\beta }{\alpha }-1)-\sigma \right]{\bar{m}}_{1}^{2}-2\frac{\beta }{\alpha }\left[(\theta +\xi ){\bar{m}}_{2}+\frac{\lambda p_{21}-p_{12}}{\alpha \lambda }\right]}{\sigma {\bar{m}}_{1}^{2}},$$

$$A_{8}=\frac{2\frac{\beta }{\alpha }\left[(\theta +\xi ){\bar{m}}_{2}+\frac{\lambda p_{21}-p_{12}}{\alpha \lambda }\right]-\left[2\frac{\beta }{\alpha }(\theta +\xi )-\sigma \right]}{\sigma {\bar{m}}_{1}^{2}},$$

$$A_{9}=\frac{2\left[(\theta +\xi ){\bar{m}}_{2}+\frac{\lambda p_{21}-p_{12}}{\alpha \lambda }\right]}{\sigma {\bar{m}}_{1}}$$

and


$$B_{3}=\sigma \beta ,\;B_{4}=\sigma \alpha {\bar{m}}_{1}.$$

Similarly, for any *w* > 0, the steady-state wealth distributions $g_{1,\infty }(w)$ and $g_{2,\infty }(w)$ always remain positive. We use MATLAB software to solve for the constants *C*_2_ and *C*_3_ that make the sum of the integrals of $g_{1,\infty }(w)$ and $g_{2,\infty }(w)$ equal to 1, ensuring that the total wealth distribution satisfies the normalization property. The solutions (27) and (28) indicate that the wealth replacement rate α/β (α/β≠1), transaction rate *θ*, and market risk *σ* determine the steady-state wealth distribution. It should be mentioned that the ratio of steady-state wealth distributions between agents in the two groups remains unchanged ($g_{2,\infty }(w)=\lambda g_{1,\infty }(w)$, and *λ* is a positive constant). This fixed ratio *λ* influences the steady-state wealth distribution. According to (3), the total steady-state wealth distribution $g_{\infty }(w)$ takes the form

$$g_{\infty }(w)=g_{1,\infty }(w)+g_{2,\infty }(w)\;=C_{2}w^{A_{4}}(B_{1}w+B_{2}{)}^{A_{5}}exp\left\{-\frac{A_{6}}{w}\right\}\;\;+C_{3}w^{A_{7}}(B_{3}w+B_{4}{)}^{A_{8}}exp\left\{-\frac{A_{9}}{w}\right\}.$$ (29)

### 4.3 Numerical experiments

#### 4.3.1 Test 1: The effects of parameters on the steady-state solution in solvable case 1.

In this section, we analyze the effects of the wealth replacement rate, trading rate and market risk on wealth distribution by drawing the graphs of the wealth density $g_{1,\infty }(w)$ and its corresponding Lorenz curves. The Lorenz curve is one of the key tools in economics for measuring wealth inequality. It provides a graphical representation of how wealth is distributed among agents in a system (see [18,22,33]). Defining $G(w)=\int_{0}^{w}g_{1,\infty }(x)dx$ as the cumulative distribution function (CDF) of the steady-state distribution $g_{1,\infty }(w)$, the Lorenz curve is expressed as


$$L(G(w))=\frac{\int_{0}^{w}g_{1,\infty }(x)xdx}{\int_{0}^{+\infty }g_{1,\infty }(x)xdx}.$$

The horizontal axis of the Lorenz curve represents the cumulative proportion of the population, ranking from those with the lowest wealth to those with the highest wealth. The vertical axis denotes the cumulative proportion of wealth held by corresponding population. Under the condition of absolute equality in wealth distribution, the Lorenz curve overlaps with the 45-degree line, which is called the line of full equality. In real economies, such perfect equality rarely exists. Thus, we utilize the degree of deviation between the Lorenz curve and the line of full equality to measure the wealth inequality. A large degree of deviation indicates a high level of wealth inequality. According to the viewpoint of Gini [39], the Gini coefficient is also a commonly used indicator for measuring the wealth distribution gap in a country or region. Its value ranges from 0 to 1, where a small value indicates a equal distribution of wealth. The calculation formula of the Gini coefficient is


$$\tilde{G}=1-2\int_{0}^{1}L(G(w))dw.$$

**Fig 2:**
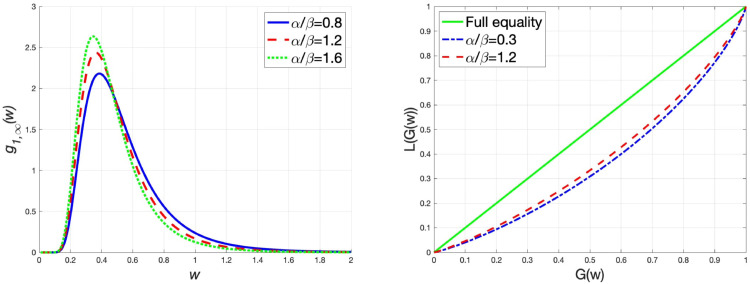
θ=0.5, ${\bar{m}}_{1}={\bar{m}}_{2}=1$, ξ=0.5 and σ=1, (left) the graph of $g_{1,\infty }(w)$ for different values of $\frac{\alpha }{\beta }$, (right) the Lorenz curves for different values of $\frac{\alpha }{\beta }$.

**Fig 3:**
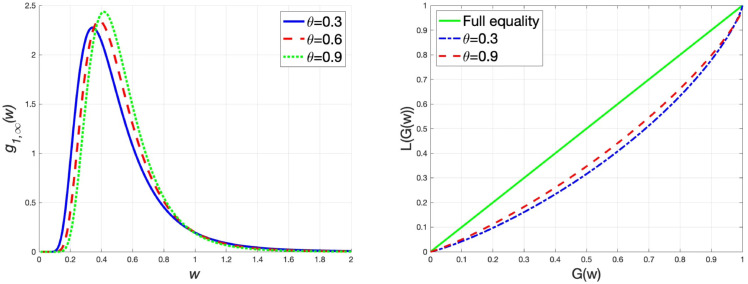
α=β=1, ${\bar{m}}_{1}={\bar{m}}_{2}=1$, ξ=0.5 and σ=1, (left) the graph of $g_{1,\infty }(w)$ for different values of *θ*, (right) the Lorenz curves for different values of *θ.*

**Fig 4:**
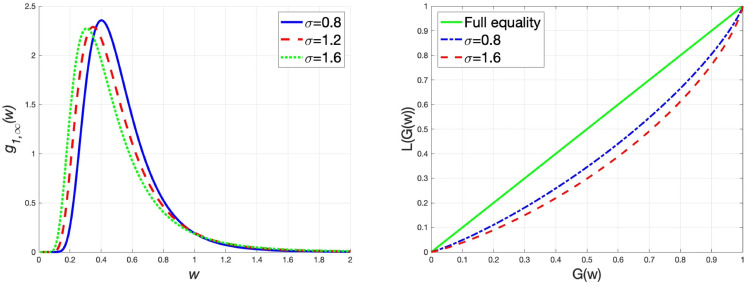
α=β=1, ${\bar{m}}_{1}={\bar{m}}_{2}=1$, ξ=0.5 and θ=0.5, (left) the graph of $g_{1,\infty }(w)$ for different values of *σ*, (right) the Lorenz curves for different values of *σ.*

Within the parameter value range, we fix the parameter values as ξ=0.5 and ${\bar{m}}_{1}={\bar{m}}_{2}=1$, and depict the graphs of the steady-state wealth distribution $g_{1,\infty }(w)$ and the Lorenz curves under different values of wealth replacement rate α/β, transaction rate *θ* and market risk *σ*, respectively. In addition, we also calculate the Gini coefficients under different parameter values to examine the changes in the wealth distribution. The results are presented in Table 2. From Fig 2, we come to the conclusion that when the wealth replacement rate of the agents increases, the steady-state wealth distribution has a thin tail, implying that the wealth distribution tends to be equal. This finding is further substantiated by examining the Lorenz curves and Gini coefficients under different values of α/β. Similarly, it is seen from Fig 3 that an increase in the transaction rate results in a thin tail of the wealth distribution, which reduces the wealth inequality. Accordingly, the Gini coefficient also decreases. In the interaction rule (4), the variance of random variables measures the market risk. As illustrated in Fig 4, the high market risk induces thick-tailed characteristics of $g_{1,\infty }(w)$, which has a negative impact on the equality of wealth distribution. Besides, rising market risk widens the deviation between the Lorenz curve and the line of full equality, indicating that market risk is detrimental to wealth equality. The Gini coefficient increases as market risk rises, which verifies this conclusion.

**Table 2:** The Gini coefficients corresponding to the wealth density at steady state under different parameter values.

α/β	Gini coefficients	*θ*	Gini coefficients	*σ*	Gini coefficients
0.3	0.2189	0.3	0.2206	0.8	0.2243
1.2	0.2035	0.9	0.1863	1.6	0.2997

#### 4.3.2 Test 2: The effects of parameters on steady-state solutions in solvable case 2.

**Fig 5:**
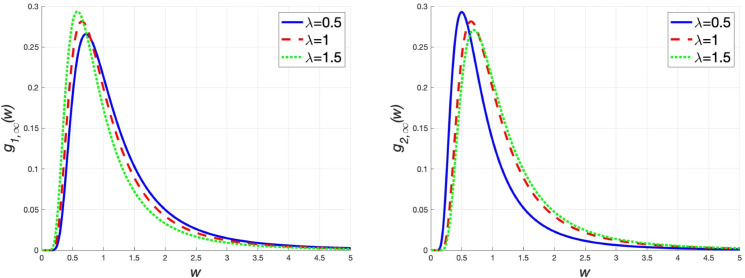
α=1, β=1, θ=0.5, ${\bar{m}}_{1}={\bar{m}}_{2}=1$, ξ=1 and σ=1. (left) The graph of $g_{1,\infty }(w)$ for different values of *λ*. (right) The graph of $g_{2,\infty }(w)$ for different values of *λ.*

The peculiarity of solvable case 2 lies in the assumption that the wealth distributions of agents in the two groups are proportional in the steady state. Namely, there is $g_{2,\infty }(w)=\lambda g_{1,\infty }(w)$. In Fig 5, we discuss the effects of the change in *λ* on the steady-state wealth distributions of agents in groups 1 and 2 (ξ=0.5, σ=1, ${\bar{m}}_{1}={\bar{m}}_{2}=1$, α=β=1 and θ=0.5). When the proportion *λ* increases (the value of $g_{2,\infty }(w)/g_{1,\infty }(w)$ increases), the tail of the steady-state wealth distribution of agents in group 1 takes on a thin form, meaning the wealth gap among agents in group 1 narrows. In contrast, the tail of the steady-state wealth distribution of agents in group 2 takes on a thick form, and the wealth gap among agents in group 2 widens. The Gini coefficients presented in Table 3 serve to verify this conclusion.

**Table 3:** The Gini coefficients corresponding to $g_{1,\infty }(w)$ and $g_{2,\infty }(w)$ under different parameter values of *λ.*

*λ*	Gini coefficients of $g_{1,\infty }(w)$	Gini coefficients of $g_{2,\infty }(w)$
0.5	0.9011	0.5450
1	0.8133	0.8133
1.5	0.6615	0.8703

## 5 Conclusions

In this paper, we employ the statistical mechanics method to investigate the wealth distribution in binary interactions between agents of two groups. We use an interaction rule with a non-zero mathematical expectation of the random variable and utilize a non-Maxwellian collision kernel. According to the exchange rule, we obtain the Boltzmann equation and transform it into the Fokker-Planck form. Under certain assumptions, we obtain the steady-state solutions in two cases. The results illustrate that the wealth replacement rate α/β, the trading rate *θ*, the market risk *σ* and the proportion *λ* affect the wealth distribution. Specifically, it is seen from the graphs of steady-state wealth distribution that the decrease of market risk *σ*, and the increases of the wealth replacement rate α/β and the trading rate *θ* lead to the improvement of wealth inequality. The above results are also verified by the Lorenz curves. Besides, improving the proportion *λ* facilitates the narrowing of wealth disparities in group 1, while simultaneously leads to a deterioration in wealth inequality of group 2.
